# MHD Free Convective Boundary Layer Flow of a Nanofluid past a Flat Vertical Plate with Newtonian Heating Boundary Condition

**DOI:** 10.1371/journal.pone.0049499

**Published:** 2012-11-15

**Authors:** Mohammed J. Uddin, Waqar A. Khan, Ahmed I. Ismail

**Affiliations:** 1 School of Mathematical Sciences, University Sains Malaysia, Penang, Malaysia; 2 Department of Engineering Sciences, PN Engineering College, National University of Sciences and Technology, Karachi, Pakistan; 3 Mathematics Department, American International University-Bangladesh, Banani, Dhaka, Bangladesh; Brandeis University, United States of America

## Abstract

Steady two dimensional MHD laminar free convective boundary layer flows of an electrically conducting Newtonian nanofluid over a solid stationary vertical plate in a quiescent fluid taking into account the Newtonian heating boundary condition is investigated numerically. A magnetic field can be used to control the motion of an electrically conducting fluid in micro/nano scale systems used for transportation of fluid. The transport equations along with the boundary conditions are first converted into dimensionless form and then using linear group of transformations, the similarity governing equations are developed. The transformed equations are solved numerically using the Runge-Kutta-Fehlberg fourth-fifth order method with shooting technique. The effects of different controlling parameters, namely, Lewis number, Prandtl number, buoyancy ratio, thermophoresis, Brownian motion, magnetic field and Newtonian heating on the flow and heat transfer are investigated. The numerical results for the dimensionless axial velocity, temperature and nanoparticle volume fraction as well as the reduced Nusselt and Sherwood number have been presented graphically and discussed. It is found that the rate of heat and mass transfer increase as Newtonian heating parameter increases. The dimensionless velocity and temperature distributions increase with the increase of Newtonian heating parameter. The results of the reduced heat transfer rate is compared for convective heating boundary condition and found an excellent agreement.

## Introduction

Conventional heat transfer fluids, for example oil, water, and ethylene glycol mixtures, are poor heat transfer fluids because of their poor thermal conductivity. Application of these fluids as a cooling tool enhances manufacturing and operating costs. Many attempts have been taken by many researchers to enhance the thermal conductivity of these fluids by suspending nano/micro particles in liquids ([1]–[2]). Nanofluids are made of ultrafine nanoparticles (<100 nm) suspended in a base fluid, which can be water or an organic solvent ([3]). Nanofluids are found to exhibit higher conductive, minimum clogging, boiling, and convective heat transfer performances compared to conventional fluids ([4]–[6]). By combining nanofluid with biotechnological components, nanotechnology can have numerous potential applications across a wide range of practical applications such as agriculture, pharmaceuticals and biological sensors. The potential forms of nanomaterials available for use in biotechnological applications includes a growing list of nanoparticles, nanowires, nanofibers, nanostructures and nanomachines ([7]). The commercialization of nanobiotechnological products seems to have a potential future and within next a few years many new products of this nature are likely to be used. Nano and micro-fluidics is a new area with potential for engineering applications, especially for the development of new biomedical devices and procedures ([8]–[9]). Napoli et al. [10] reviewed applications of nanofluidic phenomena to various nanofabricated devices related to biomolecule transport. The industrial applications of nanofluid include electronics, automotive and nuclear applications. Nanobiotechnology is also a fast developing field in many domains such as in medicine, pharmacy and agro-industry ([11]). Despite significant progress on nanofluids, variability and controversies in the heat transfer characteristics still exist ([12]–[13]). In 2010 Nasir [14], pointed out several controversial medical applications of nanofluids.

MHD flow past a flat surface has many important technological and industrial applications such as micro MHD pumps, micromixing of physiological samples, biological transportation and drug delivery ([15]–[16]). The application of the magnetic field produces Lorentz forces which are able to transport liquids in the mixing processes as an active micromixing technology method. Hence, transportation of conductive biological fluids in micro systems may greatly benefit from theoretical research in this area ([17]). Studies on MHD free convective boundary-layer flow of nanofluids are very limited. Recently, Chamkha and Aly [18] dealt with MHD free convective boundary-layer flow of a nanofluid along a permeable isothermal vertical plate in the presence of heat source or sink. They presented non-similar solutions. Nourazar et al. [19] examined MHD forced-convective flow of nanofluid over a horizontal stretching flat plate with variable magnetic field including the viscous dissipation. Very, recently Zeeshan et al. [20] investigate the MHD flow of third grade nanofluid between coaxial porous cylinders. MHD mixed convective flow of nanofluid over a stretching sheet was very recently investigated by Matin et al. [21]. As has been pointed out by others, magentic nanofluid has many applications: magnetofluidic leakage-free rotating seals, magnetogravimetric separations, acceleration/inclinations sensors, aerodynamic sensors (differential pressure, volumic flow), nano/micro-structured magnetorheological fluids for semiactive vibration dampers, biomedical applications in plant genetics and veterinary medicine.

The natural convective flow of a nanofluid past a vertical plate under different boundary condition has been investigated by several researchers ([22]–[29]. Ho et al. [30] studied natural convective flow of a nanofluid under various flow configurations. Niu et al. [31] studied slip-flow and heat transfer of a non-Newtonian nanofluid in a microtube. Kuznetsov and Nield [23] presented a similarity solution of natural convective of a nanofluid past a vertical plate. Khan and Pop [24] used the Buongiorno [32] model to study the boundary layer flow of a nanofluid past a stretching sheet. Khan and Aziz [25] also used the same model to investigate the boundary layer flow of a nanofluid past a vertical surface with a constant heat flux. Gorla and Chamkha [33] studied natural convection flow past a horizontal plate in a porous medium filled. Very recently, Aziz and Khan [34] studied natural convective flow of a nanofluid over a convectively heated vertical plate. They used the Buongiorno [32] model.

Group analysis provides a powerful, sophisticated and systematic tool for generating the invariant solutions of the system of nonlinear partial differential equations (PDEs) with relevant initial or boundary conditions. It reduces number of independent variables by one and consequently the governing PDEs are transformed into ordinary differential equations with the associated boundary conditions. Hence, it has attracted the attention of many investigators to analyze various convective phenomena subject to various flow configurations arising in fluid mechanics, aerodynamics, plasma physics, meteorology and some branches of engineering ([35]). This method has been applied by many authors in many physical problems. For example, the symmetrical properties of the turbulent boundary-layer flows were investigated by Avramenko et al. [36]. Kuznetsov et al. [37] investigated a falling bioconvection plume in a deep chamber filled with a fluid saturated porous medium theoretically. The effect of thermal radiation and convective surface boundary condition on the boundary layer flow was investigated by Hamad et al. [38]. Aziz et al. [39] studied MHD flow over an inclined radiating plate with temperature dependent thermal conductivity, variable reactive index and heat generation. Reviews for the fundamental theory of group theory to differential equations can be found in standard texts by Na [40], Ames [41], Seshadri and Na [42], Shang [43].

All of the above cited investigators applied the commonly used boundary conditions either a prescribed surface temperature (PST) or a prescribed surface heat flux (PHF), or temperature jump (TJ) or thermal convective heating (CH) (generalization of PST and TJ). There is however another class of convective flow, heat mass transfer problems where the surface heat transfer depends on the surface temperature ([44]). The situation where the heat be transported to the convective fluid via a bounding surface having finite heat capacity is known as Newtonian heating (or conjugate convective flows). Newtonian heating arise in several important engineering devices, namely in heat exchanger where the conduction in the solid tube wall is influenced by the convection in the fluid past it [45]. Other examples include conjugate heat transfer around fins where the conduction within the fin and the convection surrounding the fluid must be analyzed simultaneously to obtain important design information and convection flows setup when the bounding surfaces absorbs heat by solar radiation [44], [46]. A careful examination of literature reveals that the flow of nanofluids over a flat surface has recently received the attention of investigators because of their interesting physical characters and increasing technological and industrial applications including medical and biomedical applications.

The aim of this paper is to extend a very recent paper of Aziz and Khan [34] who studied natural convection flow due to a convectively heated vertical plate. In this paper we study magneto hydrodynamic free convection of a nanofluid over a vertical flat plate taking into account Newtonian heating boundary condition. Instead of using the existing similarity transformations in the literature, we develop similarity transformations using sophisticated group transformations method. To our best of knowledge, the present paper is the first to consider this problem so that the results are new and original. The present study find applications in cooling problems in the industry, to control the boundary layer separations and to reduce the drag etc.

## Basic Equations

**Figure 1:**
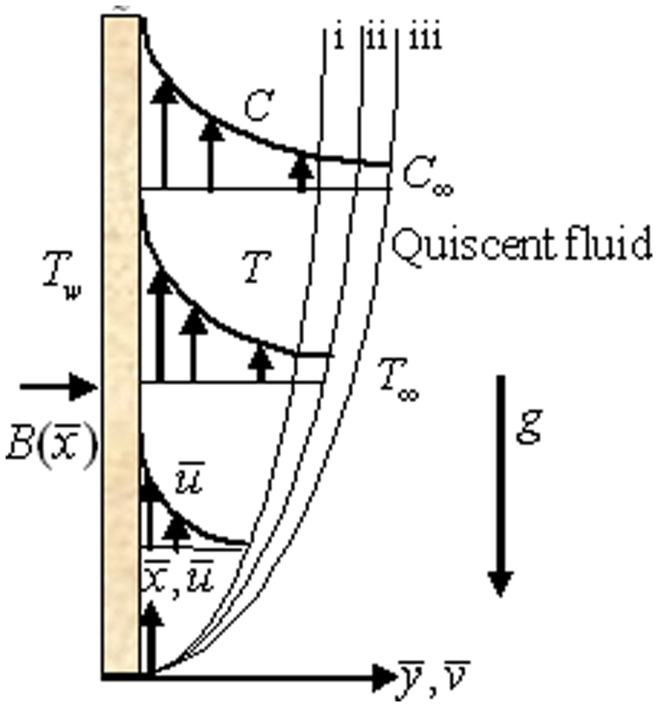
Flow configuration and coordinate system.


Consider a two dimensional steady laminar free convective boundary layer flow of a nanofluid over a permeable flat vertical plate as shown in **Fig. 1** (i, ii, iii represent momentum, thermal and nanoparticle volume fraction boundary layers). The nanoparticle volume fraction at the wall is 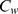. The ambient values of the temperature and nanoparticle volume fraction are denoted by 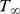 and 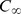 respectively. It is assumed that the surface of the plate is subject to Newtonian heating boundary condition (NH). A transverse magnetic field with variable strength 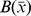 is applied parallel to the 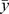 axis. It is assumed that the magnetic Reynolds number is small and hence the induced magnetic field can be neglected. The tangential and normal velocities of the fluid are respectively taken as 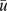 and 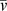. The fluid temperature and concentration are respectively denoted by 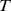 and 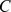. The Oberbeck–Boussinesq approximation is used. With these assumptions and the standard boundary layer assumptions, the governing equations can be written as ([34]).

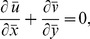
(1)

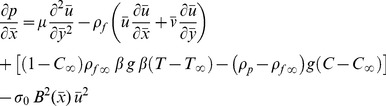
(2)

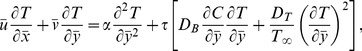
(3)

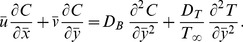
(4)subject to the boundary conditions ([47])



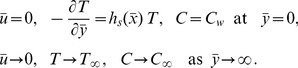
(5)where 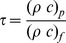 is the ratio of nanoparticle heat capacity and the base fluid heat capacity, 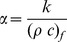 is the thermal diffusivity of the fluid, 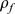 is the density of the base fluid, 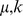 and 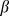 are viscosity, thermal conductivity and volumetric thermal expansion coefficient of the base fluid and 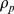 is the density of the particles, 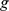 is the acceleration due to gravity, 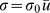 is the variable electric conductivity, 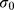 is the constant electric conductivity, 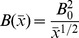 is the variable magnetic field, 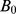 is the constant magnetic field. Here 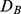 stand for the Brownian diffusion coefficient and 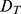 stands for the thermophoretic diffusion coefficient 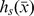 is the heat transfer coefficient. In order to compare, we shall also consider the case of convectively heated (CH) plate for which 
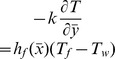
 at the boundary, 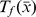 is the temperature of the hot fluid, 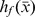 is the heat transfer coefficient.

### 2.1 Nondimensionalization

We introduce the following boundary layer variables to express Eqs. (1–5) into dimensionless form. 
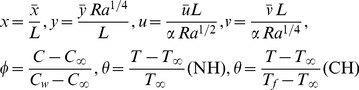
(6)where 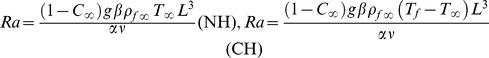 is the Rayleigh number based on the characteristic length 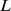. We introduce the stream function 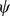 defined as 
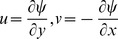
 into Eqs. (2)–(5) to reduce the number of equations and number of dependent variables. This leaves us with the following three dimensionless equations. 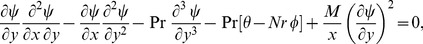
(7)

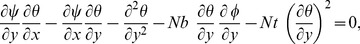
(8)

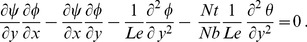
(9)Here 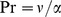 is the Prandtl number, 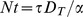 is the thermophoresis parameter, 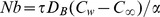 is the Brownian motion parameter, 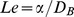 is the Lewis number, 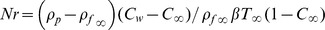 is the buoyancy ratio parameter, 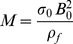 is the magnetic field parameter.

The boundary conditions become. 
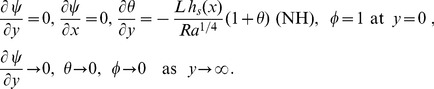
(10)along with 
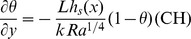
.

### 2.2 Application of Linear Group Analysis and Similarity Equations

The transported equations (7)–(10) form a highly coupled nonlinear boundary value problem. Numerical solutions of these equations are complicated and computationally expensive. Similarity solutions proved to be an efficient tool to solve various transport problems. In this section we shall show how linear group of transformations combines the two independent variables 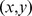 into a single independent variable 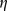 (similarity variable) and reduce Eqs. (7)–(10) into ordinary differential equations with the corresponding boundary conditions. For this purpose we scale all independent and dependent variables as. 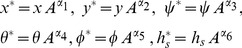
(11)where 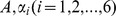 are constant ([39], [40], [43]). We seek the values of 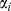 such that the form of the Eqs. (7)–(10) are invariant under the transformations. Substituting new variables in Eq. (11) into Eqs. (7)–(10), equating powers of 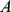 (to confirm the invariance of the Eqs. (7)–(10) under this group of transformations), we have, 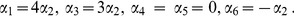
(12)


Next, we seek “absolute invariants” under this group of transformations. Absolute invariants are functions having the same form before and after the transformation.

It is clear from Eqs. (11) and (12) that. 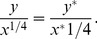
(13)


This combination of variables is therefore invariant under this group of transformations and consequently, is an absolute invariant. We denote this functional form by



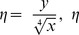
(14) is the similarity independent variable.

By the same argument, other absolute invariants are. 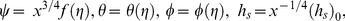
(15)where 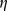 is the similarity independent variable, 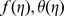 and 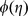 are the dimensionless velocity function, dimensionless temperature and dimensionless nanoparticle volume fraction functions respectively and 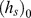 is the constant heat transfer coefficient.

Substituting Eqs. (14) and (15) into Eqs. (7)–(9), we obtain the following ordinary differential equations. 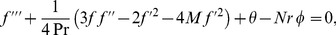
(16)

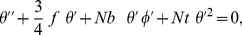
(17)

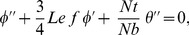
(18)subject to the boundary conditions



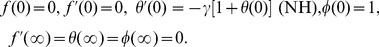
(19)along with 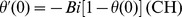


where primes denote differentiation with respect to 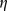. Here 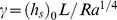 is the conjugate heat transfer parameter and 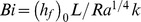 is the Biot number.

The quantities of interest, in this study, are the local Nusselt number 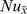 and the local Sherwood number 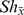 can be found from the following definition (see [48]–[50]).
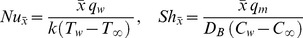
(20)where 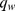, 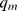 are the wall heat and the wall mass fluxes, respectively, and are defined as
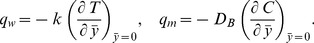
(21)


Using Eqs. (6), (14), (15), we have from Eq. (20).
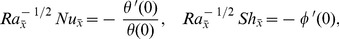
(22)where 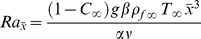 is the local Rayleigh number. In the present context, ( 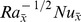 ) and ( 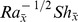 ) are referred to as the reduced Nusselt number and reduced Sherwood number (Nur and Shr), which are represented by 
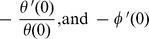


## Results and Discussion

A linear group of transformations is used to reduce the two independent variables into one and hence to reduce the governing equations into a system of non-linear ordinary differential equations with associated boundary conditions. Equations (16) to (18) with boundary conditions (19) were solved numerically using the Runge-Kutta-Fehlberg fourth-fifth order method with shooting technique. The effects of different parameters on the dimensionless flow and heat and mass transfer rates are investigated and presented graphically and compared for different thermal boundary conditions in tabular form.

### 3.1 Dimensionless Velocity Profiles

**Figure 2:**
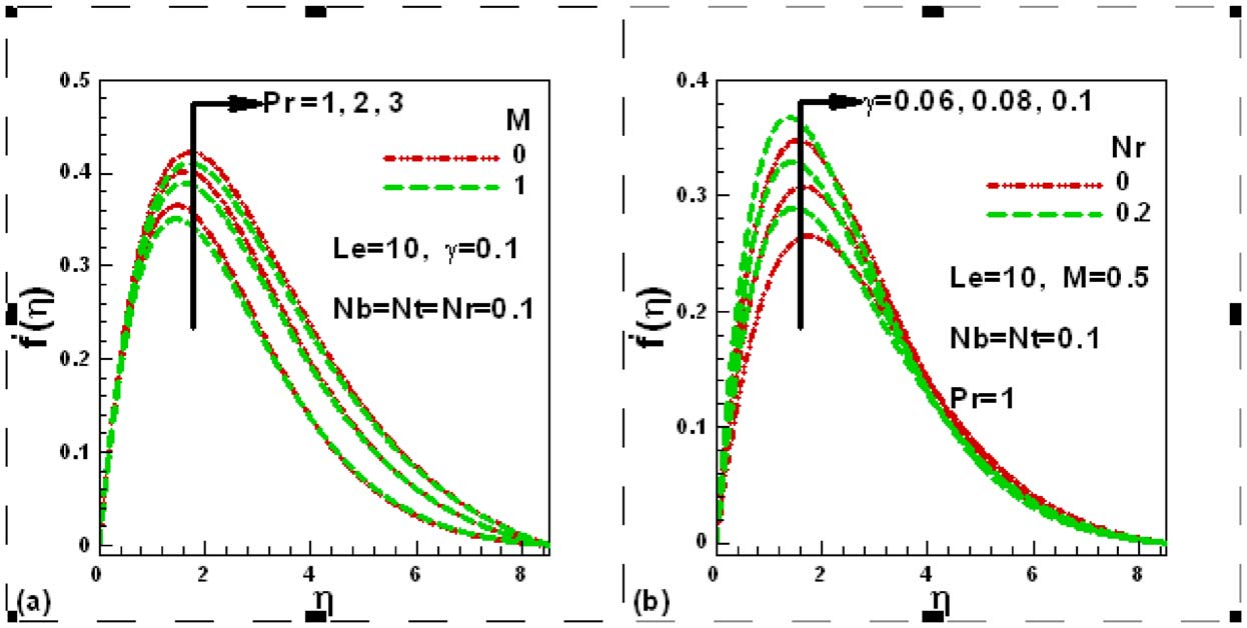
Effects of several parameters on dimensionless velocity profiles.

Figures 2 exhibit the dimensionless velocity profiles for various Prandtl numbers, magnetic field, buoyancy ratio and Newtonian heating parameters. Figure 2 (a) displays the effects of Prandtl numbers and magnetic field parameter on the dimensionless velocity. It is found that the dimensionless velocity increases with Prandtl number both for purely hydrodynamic and magneto hydrodynamic flow. It is also noticed that magnetic field reduces the dimensionless velocity for both cases. This is because application of a transverse magnetic field to an electrically conducting fluid results in a resistive-type force which tends to slow down the motion of the fluid in the boundary layer and increase the temperature and concentration within the respective boundary layers. Therefore, magnetic field is used to control boundary layer separation. Figure 2 (b) displays the effects of the buoyancy ratio and Newtonian heating parameters on the dimensionless velocity in the presence of magnetic field and nanofluid parameters. It is apparent that the dimensionless velocity rises in the boundary layer with rising of the Newtonian heating parameter both in the presence and absence of buoyancy ratio. The Newtonian heating decreases the density of nanofluid and as a result, the dimensionless velocity increases within the boundary layer. As order of buoyancy ratio increases, the velocity in the boundary layer is found to be increased.

### 3.2 Dimensionless Temperature Profiles

**Figure 3:**
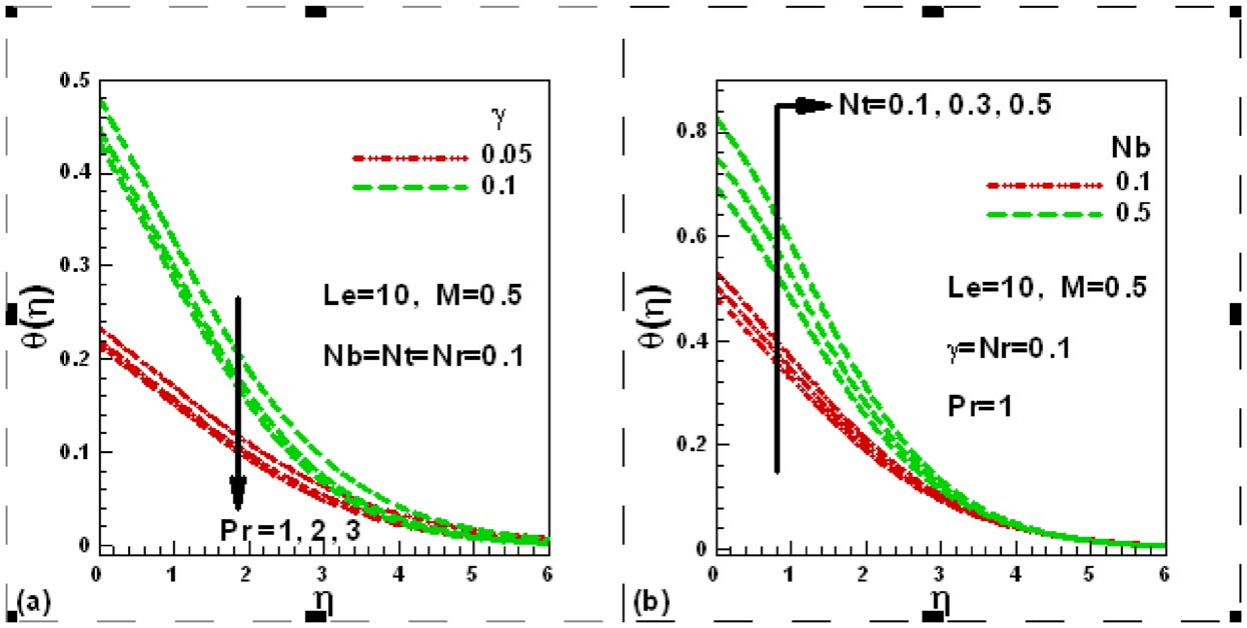
Effects of several parameters on dimensionless temperature profiles.

Figure 3(a) displays influences of Prandtl number and Newtonian heating parameter whereas Fig. 3(b) shows the effects of thermophoresis and Brownian motion parameters on the dimensionless temperature in the presence of magnetic field. The increase in Prandtl number decreases the thermal boundary layer thickness and as a result, the dimensionless temperature decreases whereas Newtonian heating increases the surface temperature of the plate. It is also evident from Fig.3 (b) that both nanofluid parameters help in increasing the surface temperature. This conclusion is in agreement with Khan and Pop [24] and Aziz and Khan [34].

### 3.3 Dimensionless Nanoparticle Volume Fraction Profiles

**Figure 4:**
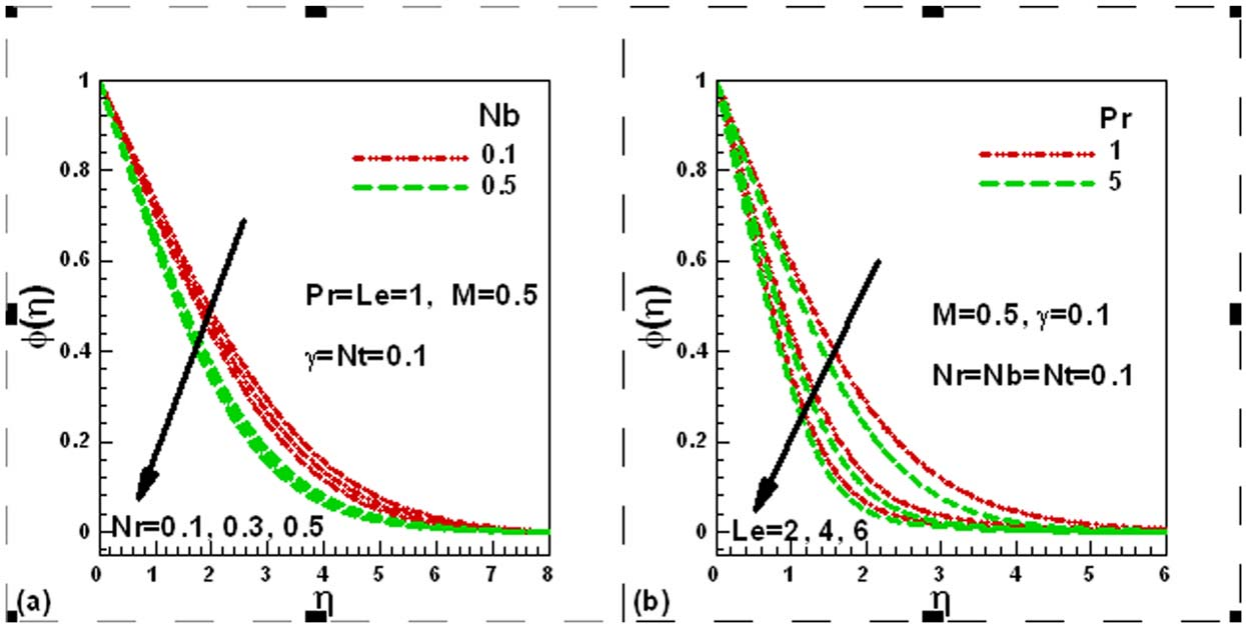
Effects of several parameters on dimensionless concentration profiles.

Figures 4 (a) and (b) illustrate the effects of the flow controlling parameters, buoyancy ratio, Brownian motion, Prandtl and Lewis numbers on the dimensionless nanoparticle volume fraction within the nanoparticle volume fraction boundary layer in the presence of magnetic field. The other parameters are kept constant. The nanoparticle volume fraction is found to reduce both with buoyancy ratio and Brownian motion parameter (Fig. 4a). A similar trend of the nanoparticle volume fraction is noticed for Prandtl and Lewis numbers as shown in Fig. 4b.

### 3.4 Dimensionless Heat Transfer Rates

**Figure 5:**
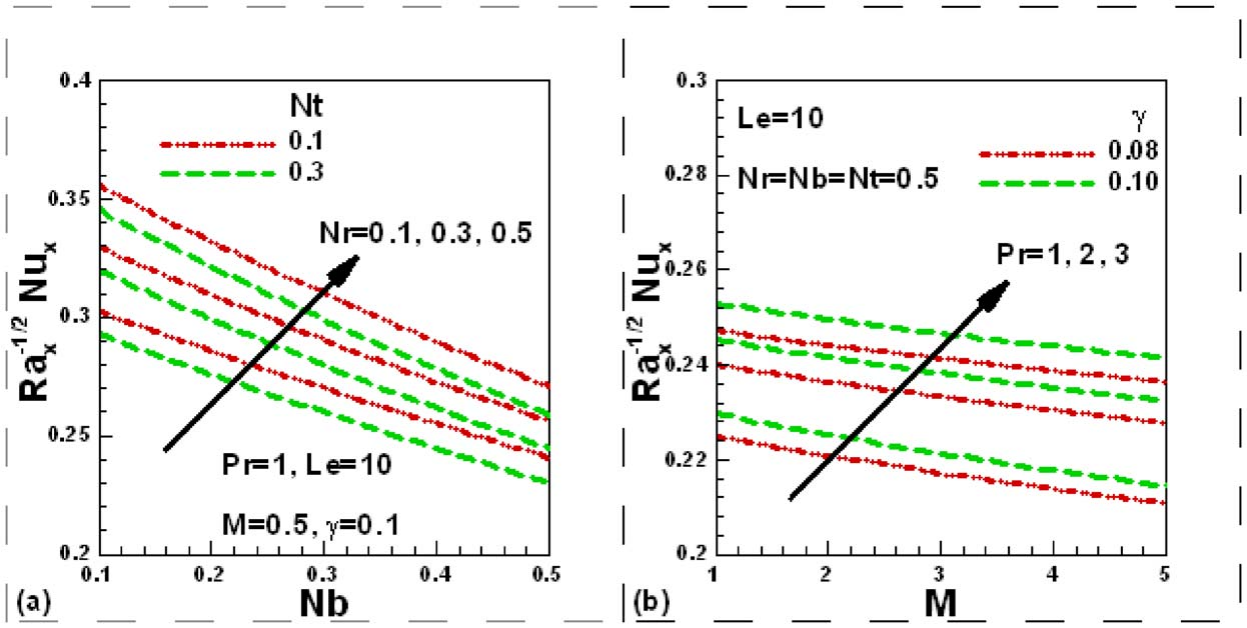
Effects of several parameters on dimensionless heat transfer rate.

In Figs. 5 (a) and (b) we present the impact of various parameters on the dimensionless local heat transfer rates. The influence of nanofluid and buoyancy ratio parameters is illustrated in Fig. 5 (a). In the presence of Newtonian heating and magnetic field, the dimensionless local heat transfer rates decrease with the Brownian motion and thermophoresis parameters, whereas they increase with an increase in buoyancy ratio parameter. Newtonian heating and Prandtl number increase the local dimensionless heat transfer rates whilst the influence of magnetic field reduces the local dimensionless heat transfer rate, as illustrated in Fig. 5 (b).

### 3.5 Dimensionless Mass Transfer Rates

**Figure 6:**
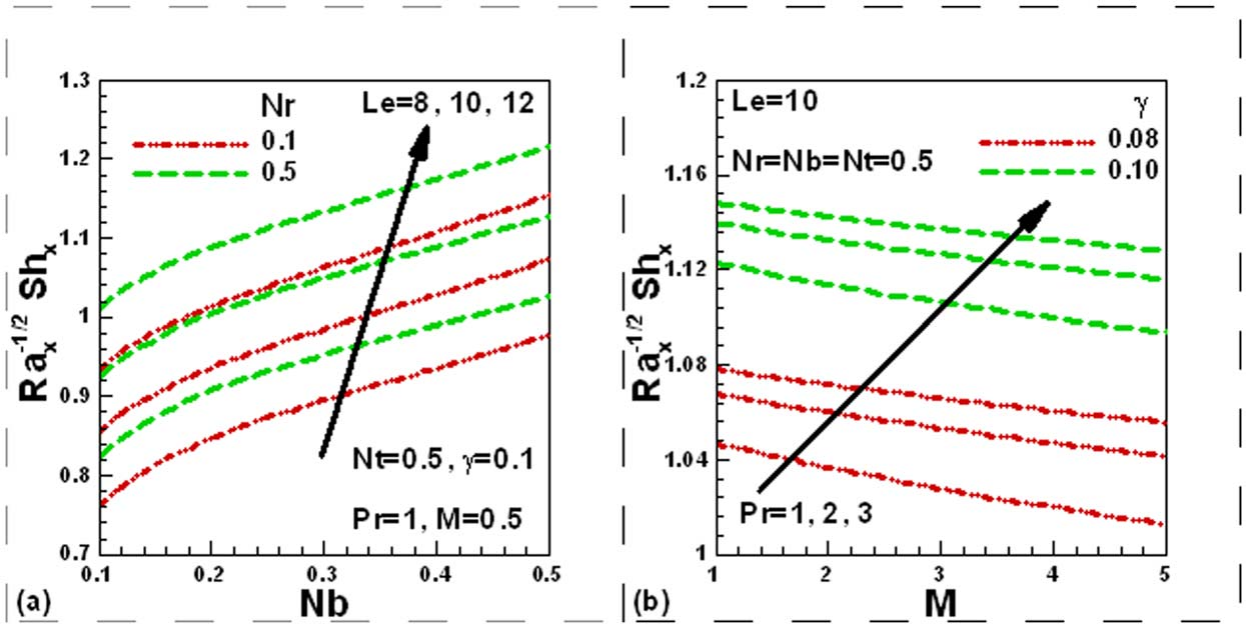
Effects of several parameters on dimensionless mass transfer rate.

The influence of the various governing parameters on the local dimensionless mass transfer rates is exhibited in Figs. 6 (a) and (b). It is clear from Fig. 6 (a) that local dimensionless mass transfer rates increase with an increase in values of the Lewis number, buoyancy ratio and Brownian motion parameters, whereas magnetic field reduces the local dimensionless mass transfer rates, as shown in Fig. 6 (b). It is also evident that an increase in the Prandtl number and Newtonian heating parameter increases the local dimensionless mass transfer rates. Finally, the reduced Nusselt numbers are compared for convective and Newtonian heating boundary conditions in Table 1 in the absence of magnetic field corresponding to different parameters. It is found that the reduced Nusselt number decreases with the buoyancy ratio and Brownian motion parameters whereas it is increased with Prandtl number. It is important to note that the reduced Nusselt numbers are higher for Newtonian heating boundary conditions than for convective boundary conditions. This conclusion is important in microelectronics industry to cool the electronic equipments.

**Table 1:** Comparison of Nusselt number values for convective and Newtonian heating when M = 0, Le = Bi = γ = 10 and Nt = 0.1.

Nb	Nr	Pr = 1		Pr = 5		Pr = 10	
		CH [34]	NH	CH[34]	NH	CH[34]	NH
0.1	0	0.34257	0.35473	0.38395	0.39928	0.3953	0.41157
	0.2	0.33659	0.36497	0.37734	0.41199	0.38856	0.42492
	0.4	0.33012	0.37477	0.37024	0.42416	0.38133	0.43771
	0.6	0.32305	0.38416	0.36252	0.43583	0.3735	0.44997
	0.8	0.31519	0.39318	0.35404	0.44704	0.36491	0.46175
0.3	0	0.2960	0.30503	0.33288	0.34434	0.34301	0.3552
	0.2	0.29178	0.31344	0.32821	0.35473	0.33826	0.36608
	0.4	0.28724	0.32147	0.32322	0.36467	0.33319	0.37651
	0.6	0.28231	0.32916	0.31785	0.37421	0.32775	0.38651
	0.8	0.27689	0.33654	0.31202	0.38338	0.32186	0.39612
0.5	0	0.2613	0.2613	0.2958	0.2958	0.30533	0.30533
	0.2	0.25777	0.26823	0.29185	0.30438	0.30131	0.31433
	0.4	0.2540	0.27486	0.28766	0.3126	0.29705	0.32294
	0.6	0.24992	0.28121	0.28318	0.32049	0.29249	0.33122
	0.8	0.24546	0.28731	0.27833	0.32809	0.28759	0.33919

### Conclusions

A two dimensional steady free convective MHD laminar incompressible boundary layer flow of an electrically conducting nanofluid past a vertical plate taking into account Newtonian heating boundary condition is studied numerically. The governing boundary layer equations are converted into highly nonlinear coupled similarity equations using linear group of transformation before being solved numerically. Based on the results, the following conclusions may be drawn:

- Increasing magnetic field strength leads to decrease the rate of heat and mass transfer rates from the vertical plate with Newtonian heating. Magnetic field significantly controls the flow, heat, and mass transfer characteristics.
- Increasing Newtonian heating parameter leads to increase the rates of heat and mass transfer.
- The velocity and the temperature distributions increase by increasing Newtonian heating parameter.
- Physical significance and application of Newtonian heating with respect to boundary layer flow problems can be found in several engineering and industrial processes as mentioned in introduction section.

The study finds application in heat exchanger where the conduction in the solid tube wall is influenced by the convection in the fluid past it. In numerous materials processing applications in mechanical and chemical engineering the fluids may be electrically conducting and as such will respond to an applied magnetic field. Such a mechanism is often used to control the heat transfer rates on various gemeotries, for example, to fine-tune the final materials to industrial specifications.
